# Thermophysiological comfort of sonochemically synthesized nano TiO_2_ coated woven fabrics

**DOI:** 10.1038/s41598-020-74357-6

**Published:** 2020-10-14

**Authors:** Muhammad Tayyab Noman, Michal Petru, Nesrine Amor, Tao Yang, Tariq Mansoor

**Affiliations:** 1 Department of Machinery Construction, Institute for Nanomaterials, Advanced Technologies and Innovation (CXI), Technical University of Liberec, Studentská 1402/2, 461 17 Liberec 1, Czech Republic; 2 Acoustic Signal Analysis and Processing Group, Faculty of Mechatronics, Informatics and Interdisciplinary Studies, Technical University of Liberec, Studentská 1402/2, 461 17 Liberec 1, Czech Republic; 3 Department of Textile Evaluation, Faculty of Textile Engineering, Technical University of Liberec, Studentská 1402/2, 461 17 Liberec 1, Czech Republic

**Keywords:** Materials for energy and catalysis, Nanoscale materials, Structural materials, Environmental sciences, Materials science, Nanoscience and technology

## Abstract

This work investigates thermophysiological comfort properties of sonochemically synthesized nano TiO_2_ coated cotton and polyester woven fabrics. The obtained results were analysed on heat and mass transfer basis. Moisture management tester and Alambeta were utilised for moisture transportation and thermal evaluation. This study precisely investigates the effects of sonication on surface roughness of nano TiO_2_ coated and uncoated samples. Ultrasonic acoustic method was applied to imbibe nano TiO_2_ on fabric samples. Surface topography, morphology and the existence of nano TiO_2_ on investigated samples were analysed by scanning electron microscopy and inductively coupled plasma atomic emission spectroscopy. In addition, standard test methods were applied to estimate physical and thermophysiological comfort properties i.e. thermal resistance, thermal diffusivity, heat flow, wetting time and accumulative one-way transport index of uncoated and nano TiO_2_ coated samples.

## Introduction

Thermophysiological comfort is one of the most demanding and desirable features of any textiles that is analysed by heat and mass transfer. Thermophysiological properties help the consumers to choose suitable garments for cold and hot weather. Clothing comfort is generally divided into various categories, however, thermophysiological comfort and sensorial comfort are the most important categories among all from experimental point of view. This work is the extension of a previously performed study about thermophysiological comfort evaluation. In a previous study, thermophysiological properties i.e. thermal conductivity, thermal absorptivity, relative water vapour permeability, evaporative resistance, air permeability and overall moisture management capacity of different fabrics (cotton and polyester) were analysed and discussed in detail^1^. In this study, thermophysiological circle is extended for thermal resistance, thermal diffusivity, maximum heat flow, wetting time and accumulative one-way transport index of nano TiO_2_ coated cotton and polyester woven fabrics. In recent years, many researchers worked on thermophysiological properties of different textiles and reported interesting results. Dalbasi and Kayseri studied thermophysiological properties of multicellular linen fabrics under various enzymatic treatments and reported that thermal conductivity is affected significantly by enzymatic treatments. In addition, enzymes treated linen shirts showed the maximum value of thermal resistance^2^. Azeem el al. studied thermophysiological properties of multifilament polyester and reported significantly higher value of thermal conductivity for nanofilament polyester than coolmax and cotton. Moreover, their results showed low thermal absorptivity for coolmax (warm feeling) and highest thermal absorptivity value for nanofilament polyester (cool feeling)^3^. Arumugam et al. studied comfort properties of 3D spacer fabrics and reported that fabric thickness is an extremely important variable for thermal conductivity and water vapour permeability. The results indicated that water vapour permeability is a function of porosity and thickness^4^. In another work, Mansoor et al. proposed a novel method to predict thermal resistance of socks in dry and wet states. They used various fibres with different combinations to develop plain socks and compared their results with thermal foot model. They reported that thermal conductivity and filling coefficient are significantly dependent on moisture content^5^. There are many other reported works on thermophysiological comfort properties of non-coated fabrics^6–10^.

Titanium dioxide (TiO_2_) is a versatile material commonly used for photocatalytic applications in textile sector^11–15^. The applications of TiO_2_ varies from sunscreens to paints and waste water treatment to self-cleaning performance^16^. Many researchers have reported successful coating of TiO_2_ on textiles for photocatalytic applications^17–23^. Sonication (utilization of ultrasonic energy) has shown its potential as a facile, economical and eco-friendly method for the fabrication of nanostructures and their deposition on textiles^24^. Acoustic cavitation is the fundamental of sonication. Ultrasonic energy induces physicochemical properties in liquids and generates infinite number of unstable bubbles that violently collapse with one another due to pressure difference and generates an excessive amount of heat with a local increase in pressure and temperature till 20 MPa and 5000 K respectively with 10^10^ Ks^−1^ cooling rate. In a previous study, a successful synthesis of nano TiO_2_ on textiles by sonication have been achieved^25^. The literature cited in above discussion indicates that very limited information is available regarding thermophysiological properties of nano TiO_2_ coated textiles. To the best of knowledge, no available literature explains the relationship between thermophysiological properties and sonochemically synthesized nano TiO_2_ coated woven fabrics. Therefore, we propose a schematic study that explicitly describes and elaborates the effects of sonication and nano TiO_2_ on thermophysiological properties of cotton and polyester woven fabrics. Furthermore, it is believed that this work is unique and extendable in its scope for other types of materials i.e. CuO and ZnO, and textile substrates.

## Materials and methods

### Materials

100% pure cotton and polyester fabrics were used throughout the study. Titanium Tetrachloride (TiCl_4_) and Isopropanol ((CH_3_)_2_CHOH) were received from Sigma Aldrich and used without any further processing.

### Physical testing

The fabrics were first conditioned for 24 h at standard conditions i.e. temperature 25 ± 2 °C and relative humidity 65 ± 2% before physical testing in accordance to standard test method ASTM D 1776-16. If a sample contains low or high humidity before testing, this test neutralizes the moisture until equilibrium achieved. Fabric mass i.e. gram per square meter (GSM) was determined by standard test method ASTM D 3776. The conditioned samples were placed in a holder and the total weight was determined for each sample. GSM was calculated by subtracting the holder weight from the total weight. Fabric thickness was measured by ASTM D 1777-96 (2019) standard method with SDL thickness meter at a pressure of 100 Pa. Samples were placed on the base of thickness gauge and the displacement among the presser foot and base was considered as sample thickness. The warp and weft yarns were made up of same materials i.e. cotton and polyester. The detail of the constructional parameters of used fabric samples is presented in Table 1.

**Table 1:** Constructional parameters of used fabrics in detail.

Sample ID	Composition (100%)	Weave	Yarn count (tex)	Yarn density (threads/inch)	TiO_2_ amount (ppm)	GSM (gm^−2^)	Thickness (mm)
S_1_	Cotton	Plain	22	74	–	110	0.25
S_2_	Cotton	Plain	22	74	355	113	0.29
S_3_	Cotton	Plain	22	74	990	116	0.31
S_4_	Cotton	Plain	28	52	–	224	0.66
S_5_	Cotton	Plain	28	52	370	227	0.68
S_6_	Cotton	Plain	28	52	965	230	0.70
S_7_	Polyester	Plain	20	80	–	118	0.32
S_8_	Polyester	Plain	20	80	401	122	0.35
S_9_	Polyester	Plain	20	80	972	125	0.38
S_10_	Polyester	Plain	27	58	–	230	0.66
S_11_	Polyester	Plain	27	58	395	232	0.75
S_12_	Polyester	Plain	27	58	985	235	0.79

### Simultaneous synthesis and coating of nano TiO_2_ on fabrics

The simultaneous synthesis and coating of nano TiO_2_ on both fabrics were achieved by a method as reported in a previous study^12^. In a typical process, fabric samples were immersed in glass beakers containing TiCl_4_, distilled H_2_O and isopropanol. The suspension was then sonicated under Bandelin Sonopuls HD 3200 ultrasonic system with 20 kHz frequency, 200 W input power and 50% efficiency for 1 h to complete the reaction mechanism. The schematic illustration of proposed process is presented (see the Supporting Information). The deposition of nano TiO_2_ on samples, surface topography and morphology were analysed by ultrahigh-resolution scanning electron microscopy (UHR-SEM), from Carl Zeiss. Energy dispersive X-ray (EDX) spectrophotometer was utilised to estimate elemental percentage on samples surface. X-ray diffractometry (XRD) analysis was performed by an X’Pert PRO X-ray diffractometer under Cu Kα radiation at wavelength λ = 0.15406 nm and scanning angle (2θ) range 10°–70°. The deposited amount of nano TiO_2_ on samples was calculated by inductively coupled plasma atomic emission spectroscopy (ICP-AES).

### Thermophysiological comfort properties

For thermal resistance (R) [m^2^ KW^−1^], thermal diffusivity (a) [m^2^ s^−1^] and transient heat flow (Q) [Wm^−2^], Alambeta by Sensora, Czech Republic was used. Alambeta measures the thermal properties of a sample both in transient and steady states. The working principle is based on coefficient of thermal conductivity that computes the net amount of heat passes from a material having area of 1 m^2^ within 1 s and covers a distance of 1 m with temperature difference of 1 K. Thermal resistance (R) is measured by given equation.

1 $$R = {\raise0.7ex\hbox{$h$} \!\mathord{\left/ {\vphantom {h \lambda }}\right.\kern-\nulldelimiterspace} \!\lower0.7ex\hbox{$\lambda $}} $$

In Eq. 1, $\lambda$ represents coefficient of thermal conductivity and *h* is fabric thickness. Thermal diffusivity (a) is a measure of heat flow along with fabric thickness in a perpendicular direction to surface area. Thermal diffusion is a transient thermal parameter that is precisely associated with two other important thermal comfort parameters i.e. thermal conductivity and thermal absorptivity. Transient parameters are calculated when the fabric gets a real contact with the body or the skin. Thermal diffusivity is calculated by Eq. 2.

2 $$a = \left( {{\raise0.7ex\hbox{$\lambda $} \!\mathord{\left/ {\vphantom {\lambda  b}}\right.\kern-\nulldelimiterspace} \!\lower0.7ex\hbox{$b$}}} \right)^{2}  $$

In above equation, $\lambda$ and *b* represent the coefficient of thermal conductivity and thermal absorptivity. Another important thermal parameter measured by Alambeta is heat flow. When a body is in contact with the fabric in the absence of wind flow and body movement, Alambeta senses the flow of heat from body to fabric.

Moisture transportation properties i.e. wetting time and accumulative one-way transport index were measured by moisture management tester (MMT). Standard test method (AATCC 195-2009) was used to investigate moisture transportation. In MMT, wetting time (top and bottom) is considered as a time when surface (top and bottom) of fabric sample begins to wet accordingly. However, accumulative one-way transport index is the difference between area of moisture content curves of a sample for top and bottom surfaces with respect to time.

### Statistical analysis

Regression analysis and analysis of variance (ANOVA) were applied to test the significance and the effectiveness of selected variables on thermophysiological properties of woven fabrics.

## Results and discussions

### SEM, EDX and XRD analysis

**Figure 1:**
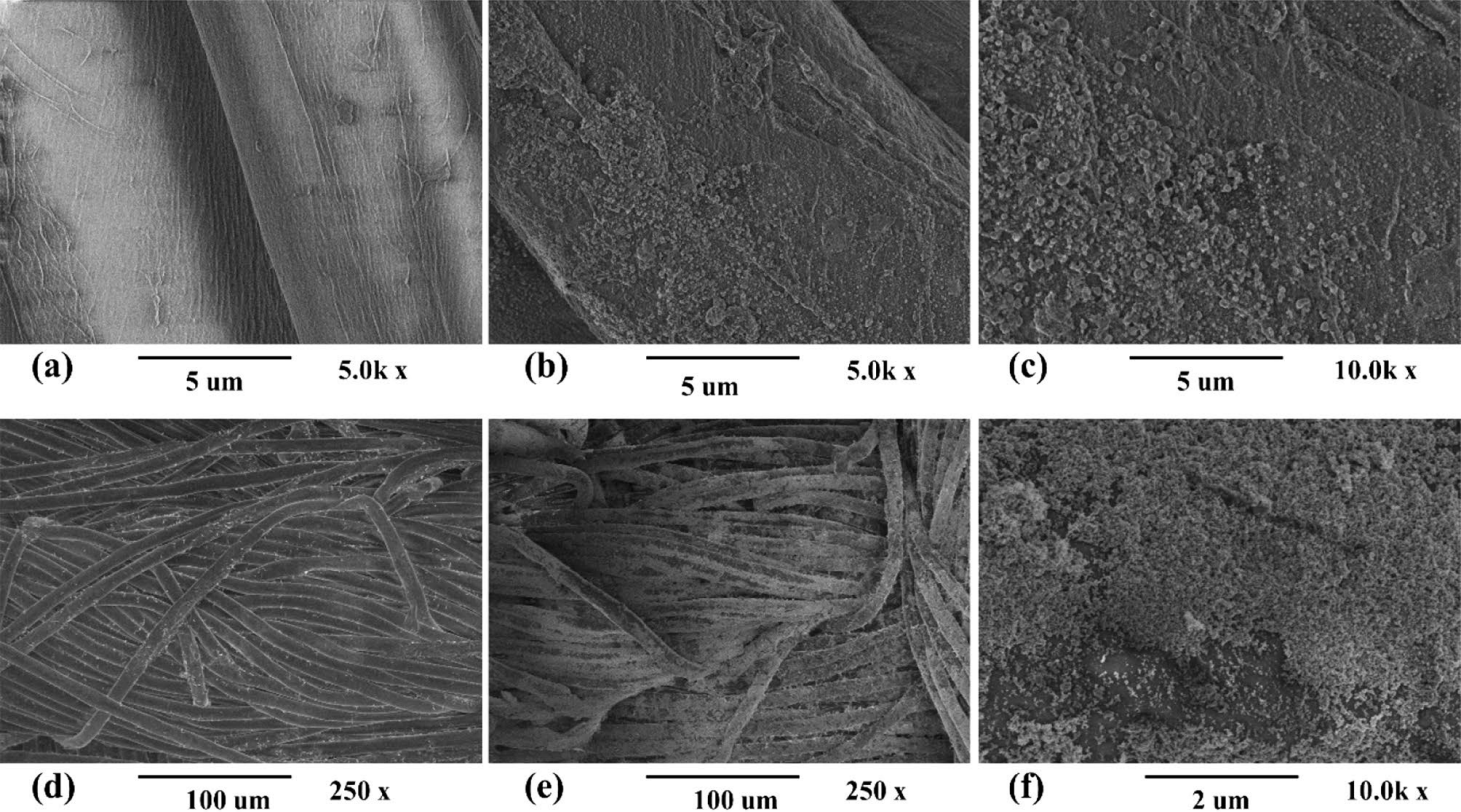
SEM images of cotton samples (**a**) S_1_ (untreated), (**b**) sample S_3_, and (**c**) higher magnification of S_3_, and SEM photographs of polyester samples (**d**) S_10_ (untreated), (**e**) sample S_12_, and (**f**) higher magnification of S_12_.

Figure 1 illustrates the results of surface topography and morphology of uncoated and nano TiO_2_ coated samples. SEM images were taken at magnification 5.0 k, 10.0 k, 250 × and 10.0 k for nano TiO_2_ coated cotton and polyester samples respectively. Figure 1a and d show very smooth and clean surfaces of uncoated samples (untreated). Figure 1c and f were taken at higher magnification to visually evaluate the coated amount (percentage) of nano TiO_2_ on samples. A quasi-spherical shape of nano TiO_2_ and homogenous distribution on both substrates was observed as described (Fig. 1b,c,e,f). It was found that the entire surface is being covered by nanoparticles due to longer sonication process and particles are attached with surface as a thick smooth layer and overwhelmingly aggregated.

Moreover, EDX and XRD analysis were carried out in order to detect the elements, their composition and their weight percentage in the developed samples as well as to detect the purity of the crystalline phase respectively and the results are illustrated and discussed (see the Supporting Information).

### Inductively coupled plasma atomic emission spectroscopy (ICP-AES)

ICP-AES study confirmed the presence of nano TiO_2_ on all coated samples i.e. S_2_, S_3_, S_5_, S_6_, S_8_, S_9_, S_11_ and S_12_, and absence on uncoated samples. The characteristic peak of Ti element was counted as observed in emission spectra to measure the coated amount on all samples and reported in Table 1. The coated amount of nano TiO_2_ was 990 ppm, 965 ppm, 972 ppm and 985 ppm for samples S_3_, S_6_, S_9_, and S_12_ respectively.

### Thermophysiological properties

Table 2 represents the experimental results of thermophysiological properties i.e. thermal resistance, thermal diffusivity, heat flow, wetting time and accumulative one-way transport index of all samples. The results were discussed one by one in this section. This discussion is a depiction that thermophysiological comfort is a function of fabric thickness and nano TiO_2_ coated amount on samples. Furthermore, regression method was performed to evaluate the influential tendency of variables and their dependency strength. A linear regression equation with coefficient of determination (R^2^) was derived individually for all properties.

**Table 2:** The overall thermophysiological comfort properties of used woven fabrics.

Sample ID	Thermal resistance (R) [m^2^ KW^−1^]	Thermal diffusivity (a) [m^2^ s^−1^]	Heat flow [Wm^−2^]	Wetting time [s]	Accumulative one-way transport index [%]
S_1_	17.4	0.092	0.431	11.607	160.2892
S_2_	14.3	0.056	0.547	6.645	438.7274
S_3_	12.1	0.038	0.597	2.901	523.5891
S_4_	9.7	0.075	0.479	20.873	297.7282
S_5_	8.1	0.048	0.589	10.951	471.4023
S_6_	6.9	0.033	0.646	5.769	583.3221
S_7_	15.1	0.051	0.516	27.818	43.6503
S_8_	13.2	0.043	0.598	6.740	453.0567
S_9_	10.1	0.031	0.654	2.187	617.4924
S_10_	8.1	0.046	0.557	51.325	46.7986
S_11_	5.3	0.039	0.699	12.296	523.3271
S_12_	3.9	0.026	0.784	4.390	741.1932

#### Thermal resistance

**Figure 2:**
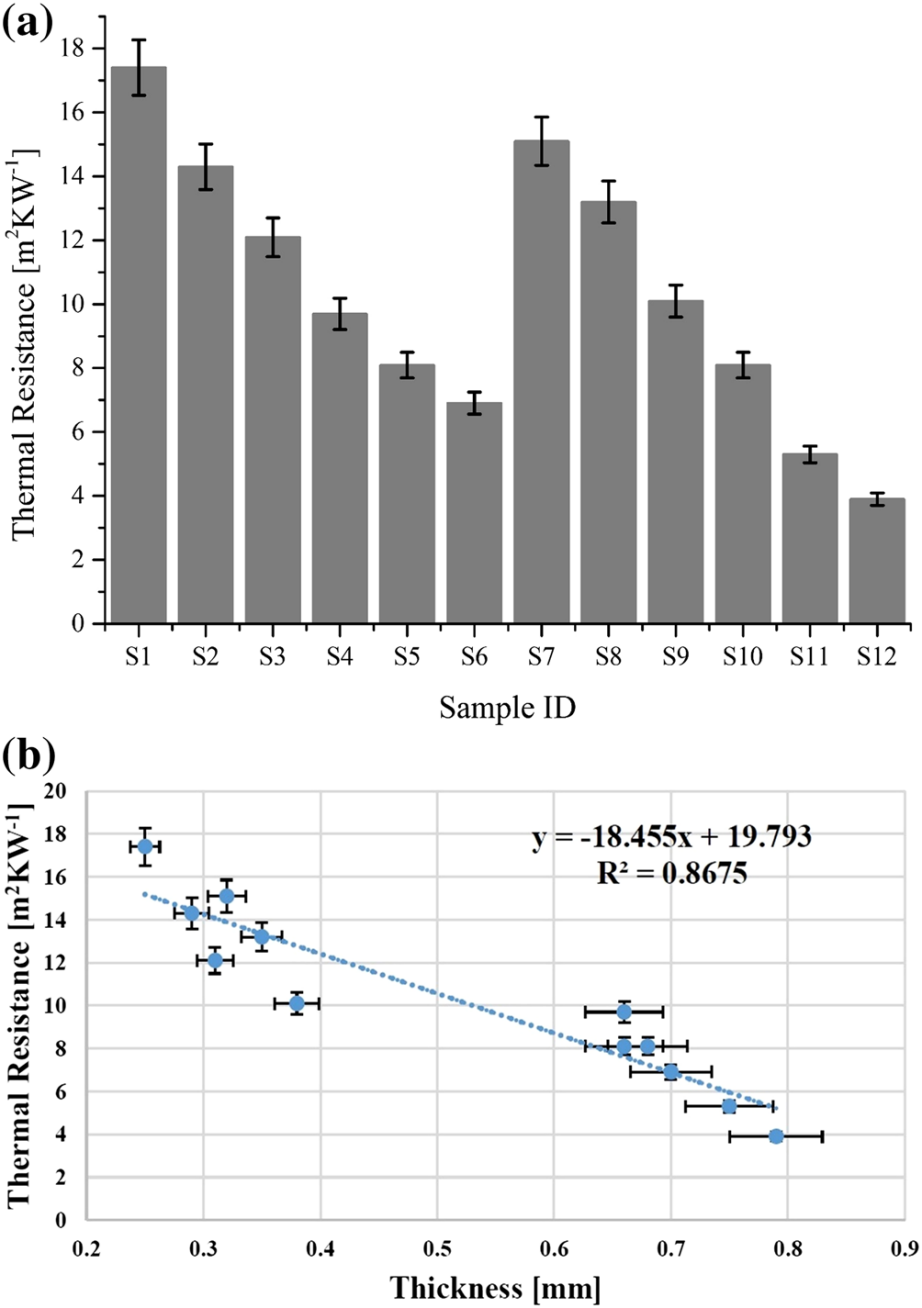
(**a**) Thermal resistance of all tested samples for cotton (S_1_ to S_6_) and polyester (S_7_ to S_12_) fabrics. (**b**) Thermal resistance of used woven fabrics as a function of thickness.

In textiles, thermal resistance is considered as one of the most influential, significant and important comfort evaluation criteria. Thermal resistance reflects the capability of a fabric to prevent heat flow from one side to another at a given unit area. A lower value of thermal resistance indicates a significantly higher amount of heat transfer from our body to fabric and vice versa. The results regarding thermal resistance of all fabric samples are presented in Fig. 2a. Thermal resistance of uncoated cotton samples (S_1_ and S_4_) and uncoated polyester samples (S_7_, S_10_) was significantly higher than nano TiO_2_ coated ones i.e. (S_2_, S_3_, S_5_, S_6_) for cotton and (S_8_, S_9_, S_11_, S_12_) for polyester. The results are quite interesting as thermal resistance increases with an augmentation in thickness. However, there are many other parameters that remain constant i.e. structure of the fabric. In this study, the impact of sonication on structure and surface of woven fabrics was investigated. The obtained results illustrate that applied treatment reduced air gaps inside the fabric structure and hence induced a positive effect on the structure and surface properties of used textiles. In addition, the results depict that nano TiO_2_ coating covered the void spaces on the surface of cotton and polyester fabrics and results in the reduction of air trapped inside the fibre volume, therefore, a decrease in thermal resistance of all coated samples was observed. The results also explain that cotton fabric samples showed less reduction in thermal resistance than polyester which means that the applied treatment improves the thermal conductivity of polyester more than cotton. Therefore, the overall results conclude a more significant effect of applied method on polyester than cotton. The obtained results are supported by a previous investigation of Gunasekaran et al.^26^.

Figure 2b shows thermal resistance results of all samples as a function of thickness. Before discussion and for better understanding of this thematic study, a key point should be noted that fabric thickness is a function of nano TiO_2_ coated amount on fabrics. This means thermophysiological properties are directly related to nano TiO_2_ coated amount and this coated amount is the outcome of prolonged sonication time. So, only thickness related results are elaborated and discussed for thermophysiological properties of nano TiO_2_ coated samples. The trendline shows a decreasing tendency for thermal resistance with an augmentation in thickness as presented in Fig. 2b. The R^2^ coefficient and regression equation statically describe thermal resistance dependency on fabric thickness. A strong negative linear relationship with a strong dependency trend was observed between thickness and thermal resistance.

#### Thermal diffusivity

**Figure 3:**
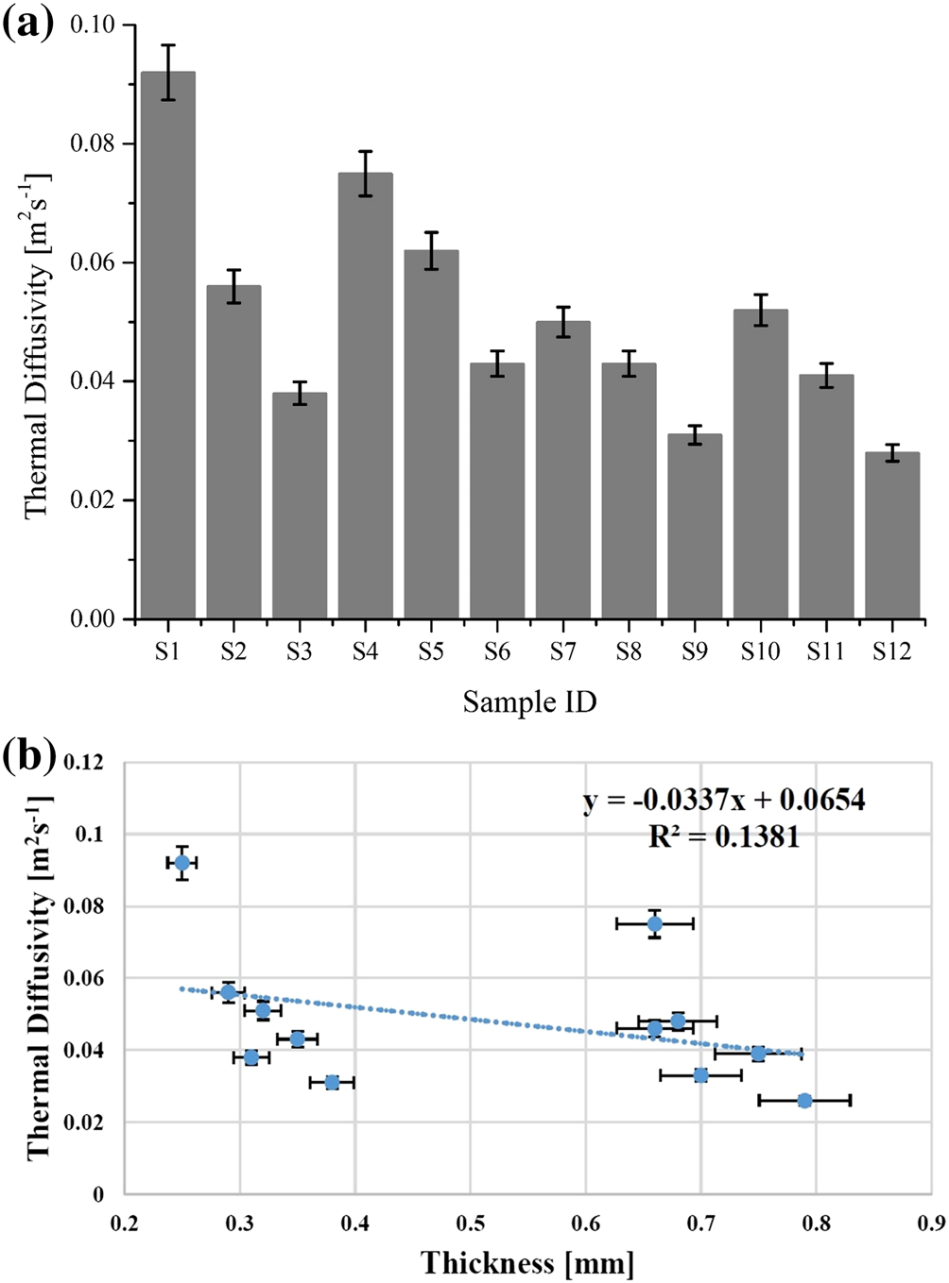
(**a**) Thermal diffusivity of all tested samples for cotton (S_1_ to S_6_) and polyester (S_7_ to S_12_) fabrics. (**b**) Thermal diffusivity of used woven fabrics as a function of thickness.

Thermal diffusivity is another influential and transient thermal parameter and a subject of great interest as it is attributed with thermal conductivity and absorptivity. Thermal diffusivity has an inverse relationship with thermal absorptivity as described in Eq. 2. Therefore, a higher thermal diffusivity value gives warmer feeling when the skin gets in touch with fabric. Thermal diffusivity results of all samples are shown in Fig. 3a. The experimental values of thermal diffusivity for all nano TiO_2_ coated samples were lower than uncoated samples of cotton (S_1_ and S_4_) and polyester (S_7_ and S_10_) respectively. The results confirmed that all nano TiO_2_ coated samples provide cool feeling on touch. The obtained results are supported by a previous investigation of Varshney et al.^27^.

Figure 3b shows thermal diffusivity results of all samples as a function of thickness. The trendline illustrates a decreasing tendency for thermal diffusivity with an augmentation in fabric thickness. The R^2^ coefficient and regression equation explain thermal diffusivity dependency on fabric thickness. A negative linear relationship with a dependency trend was found between thermal diffusivity and thickness. A lower value of R^2^ indicates a random distribution of the experimental values and the presence of an outlier.

#### Heat flow

**Figure 4:**
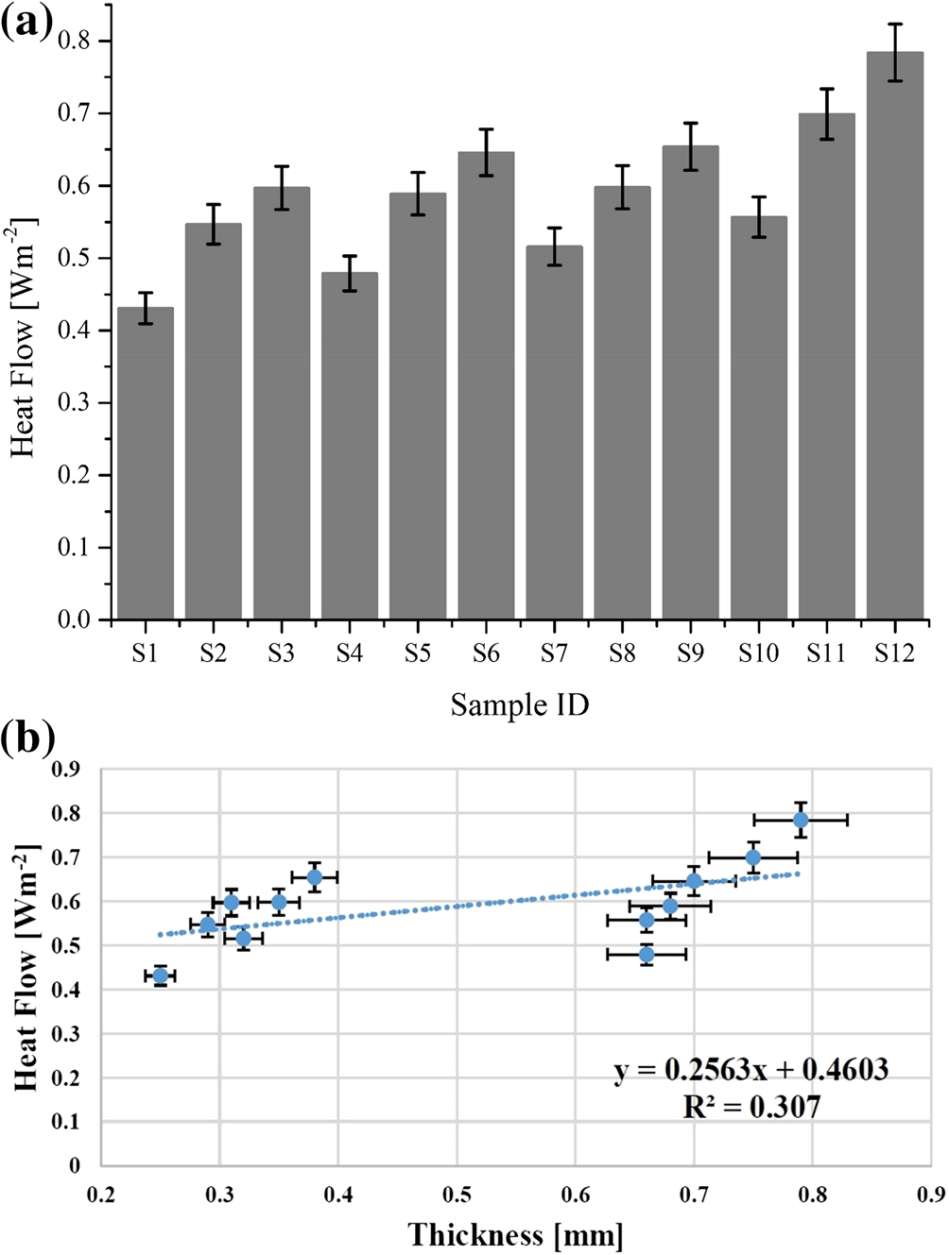
(**a**) Heat flow result of all tested samples for cotton (S_1_ to S_6_) and polyester (S_7_ to S_12_) fabrics. (**b**) Heat flow evaluation of used woven fabrics as a function of thickness.

The quantity of transient heat flow (Q) is calculated in terms of peak heat flow and stationary heat flow i.e. *q*_*max*_ and *q*_*s*_ respectively. Gradually, the value of peak heat flow decreases and stabilise at stationary heat flow. Theoretically, an augmentation in heat flow results an increment in thermal conductivity of the fabric and vice versa. The results of transient heat flow of all samples are illustrated in Fig. 4a. The results of transient heat flow for all nano TiO_2_ coated samples were higher than uncoated samples of cotton (S_1_ and S_4_) and polyester (S_7_ and S_10_) respectively. The results confirmed that an augmentation in fabric thickness due to applied treatment increased the amount of heat flow for nano TiO_2_ coated fabrics. In comparison, polyester fabric samples showed higher results for heat flow than cotton fabric samples. The obtained results are supported by a previous investigation of Dalbasi and Kayseri^2^.

Figure 4b explains the results of heat flow as a function of thickness. The trendline displays an increasing tendency of transient heat flow with an augmentation in fabric thickness. The R^2^ coefficient and regression equation explain heat flow dependency on fabric thickness.

#### Wetting time

**Figure 5:**
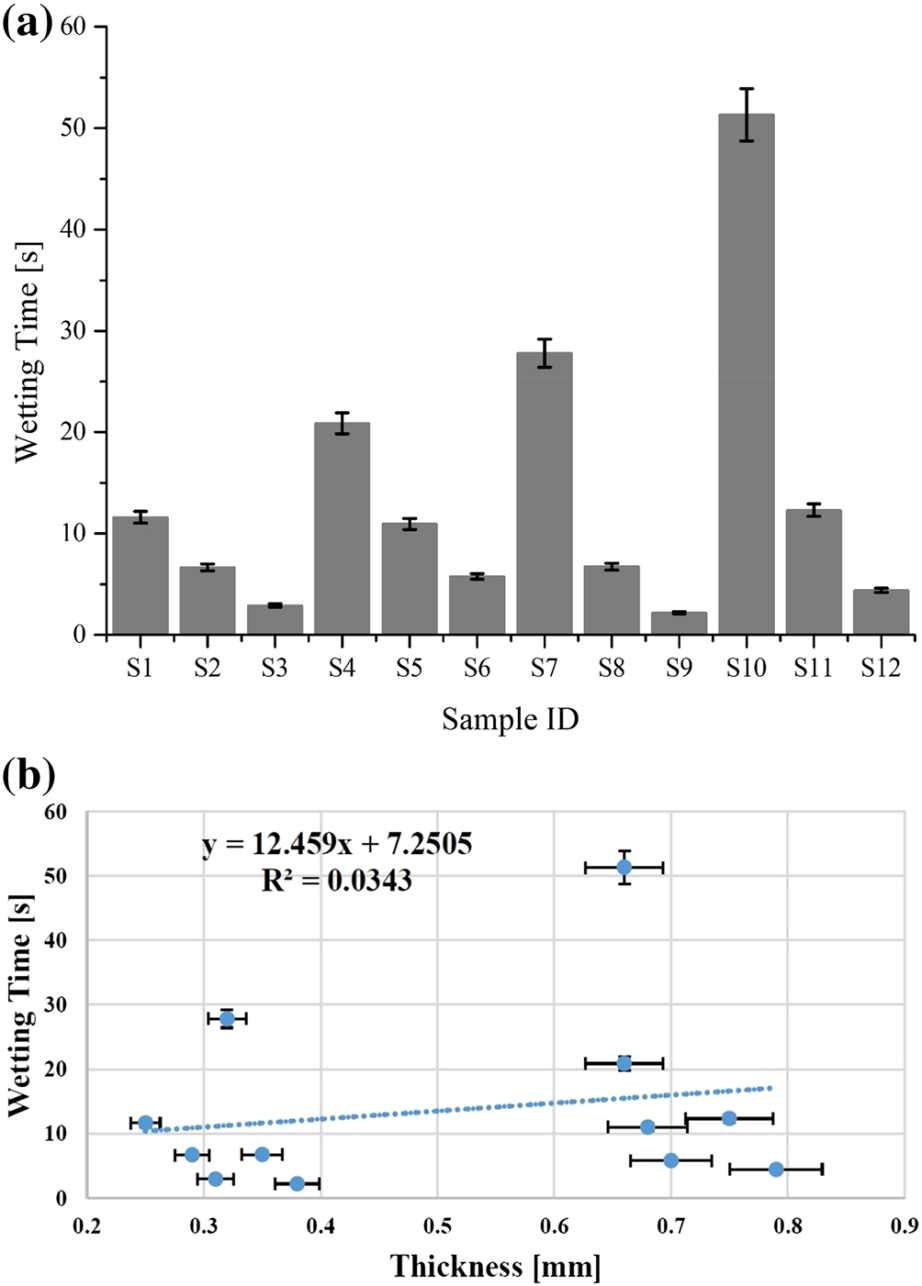
(**a**) Wetting time of all tested samples for cotton (S_1_ to S_6_) and polyester (S_7_ to S_12_) fabrics. (**b**) Wetting time of used woven fabrics as a function of thickness.

Liquid transportation behaviour of selected fabrics was studied by two crucial parameters i.e. wetting and accumulative one-way transport index. For wetting, top wetting time was considered a threshold to understand the nature of the fibre used in fabric construction. In general, hydrophobic fibres take longer time for wetting. The wetting time for all samples was evaluated and presented in Fig. 5a. The results show that wetting time for all nano TiO_2_ coated samples was lower than uncoated samples of cotton (S_1_ and S_4_) and polyester (S_7_ and S_10_) respectively. These results depict that coating of nano TiO_2_ by sonication (applied treatment) induced positive effects on hydroscopic nature of used textiles and increased their hydrophilicity to a significant level. Moreover, these results explain the role of sonication for an augmentation of thermophysiological comfort of woven fabrics. These results are supported by a previous investigation of Karthik et al.^28^.

Figure 5b shows that wetting time is a function of thickness and the trendline displays an increasing tendency of wetting time with an augmentation in fabric thickness. The R^2^ coefficient and regression equation explain the dependency of wetting time on fabric thickness. A random distribution of the experimental values and the presence of an outlier lowered the value of R^2^ coefficient.

#### Accumulative one-way transport index

**Figure 6:**
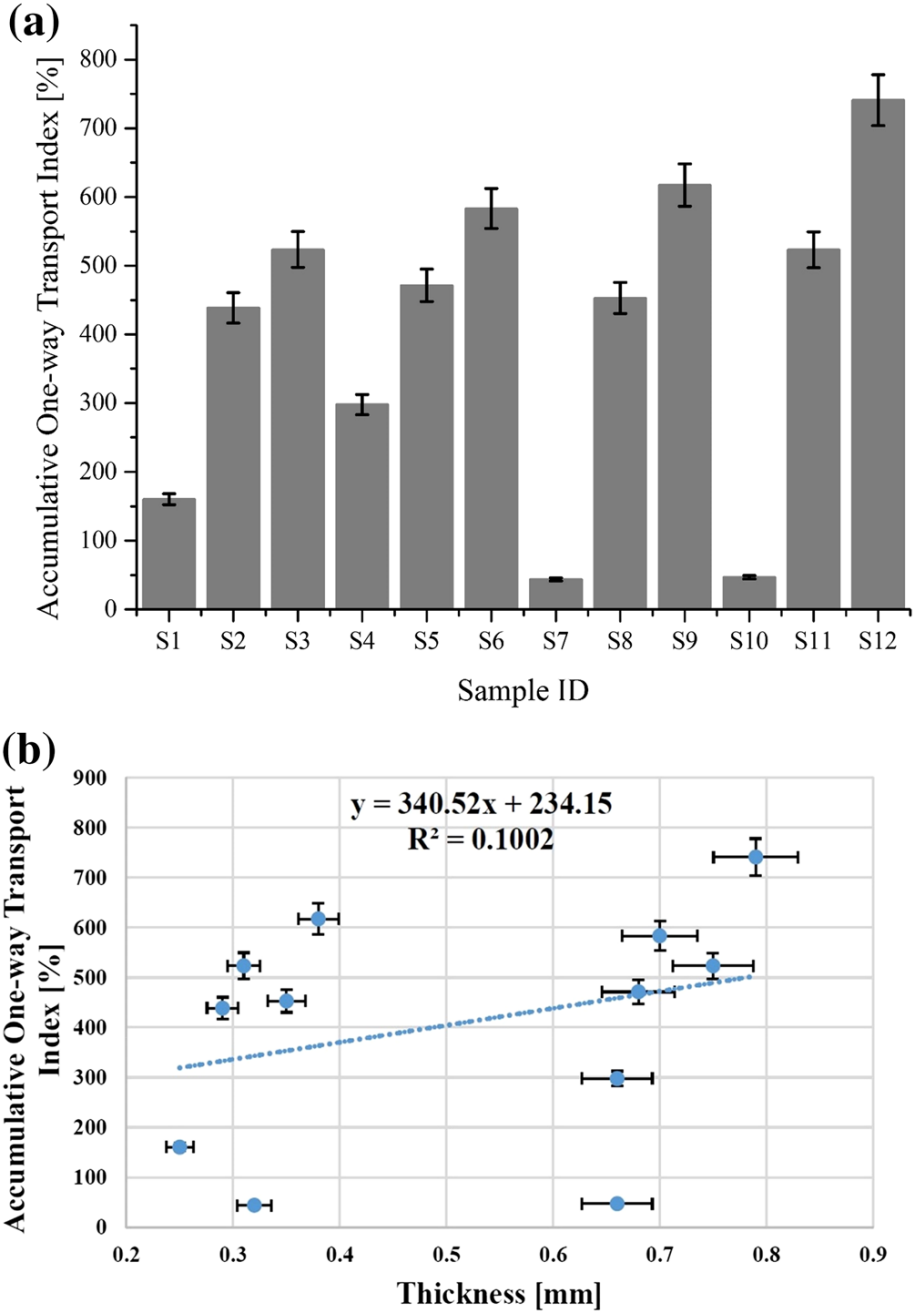
(**a**) Accumulative one-way transport index of all tested samples for cotton (S_1_ to S_6_) and polyester (S_7_ to S_12_) fabrics. (**b**) Accumulative one-way transport index of used woven fabrics as a function of thickness.

Another important indicator that reflects the overall thermophysiological comfort of textiles to a great extent is accumulative one-way transport index. By definition, accumulative one-way transport index is the difference between the area of moisture content curve among top and bottom surfaces of a sample with respect to time. The results achieved for accumulative one-way transport index of all samples are illustrated in Fig. 6a. The results proposed a significant augmentation in the values of transport index for all nano TiO_2_ coated samples than uncoated samples of cotton (S_1_ and S_4_) and polyester (S_7_ and S_10_) respectively. The results reveal that coating of nano TiO_2_ on both fabrics by sonication significantly improved moisture transportation properties. The acceleration of fluid flow during sonication and liquid penetration inside the fibre internal structure as well as fibre swelling due to cavitation results in better moisture management properties^12^.

Figure 6b shows the results of accumulative one-way transport index as a function of thickness. The trendline shows a prominent increase in accumulative one-way transport index values with an augmentation in fabric thickness. Moreover, R^2^ coefficient and regression equation explain the dependency of one-way transport index on fabric thickness. A random distribution was observed for the results of accumulative one-way transport index. These results are supported by a previous investigation of Angelova et al.^6^.

**Figure 7:**
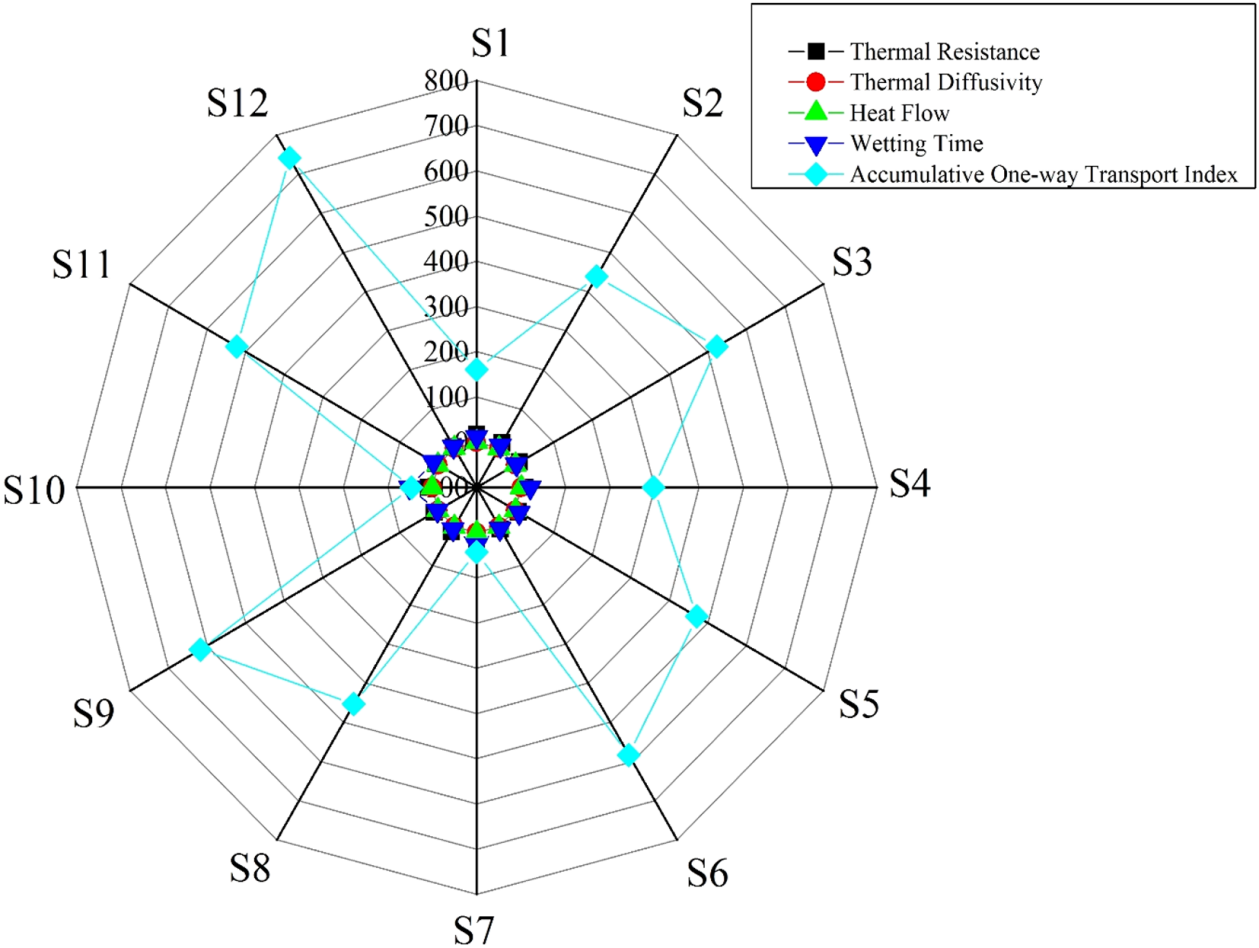
Spider plot for a twinkling comparison of investigated thermophysiological properties of nano TiO_2_ coated samples.

The results collectively propose that applied treatment (coating of nano TiO_2_ on both fabrics by sonication) induced positive physicochemical changes and enhanced thermophysiological comfort properties of woven textiles. The benefits of sonication for the synthesis of nanomaterials were explained thoroughly in previous studies^23,25^. A twinkling comparison for investigated thermophysiological properties of all samples is presented in a spider plot based on the original experimental values and illustrated in Fig. 7.

### Analysis of variance (ANOVA)

Analysis of variance (ANOVA) is an important tool to examine the effectiveness of various parameters, the interaction of data between variables and observed responses, the accuracy and the repeatability of the experiments. The results were used to judge the goodness of fit for all variables with respect to relevant response. The results demonstrate that the designed ANOVA model for thermal resistance was statistically significant for F-value of 73.899 and p-value prob > F of 0.0000036 as shown in Table 3. R^2^ coefficient was used to analyse the fit of the model. The results explained that only 3.49% of the total variables cannot be explained by the designed model for thermal resistance of all samples.

**Table 3:** ANOVA results for thermal resistance.

Source	df	SS	MS	F value	p-value prob > F	Remarks
Model	3	182.098	60.699	73.899	0.0000036	Significant
Residual	8	6.571	0.821			
Total	11	188.67				

R-squared: 0.9651, adjusted R-squared: 0.9521.

ANOVA results for thermal diffusivity were significant (F-value 5.221) and prob > F (p-value 0.0274) as described in Table 4. The results of R-squared coefficient explained that 33.81% of the total variables cannot be explained by the designed model for thermal diffusivity of used samples.

**Table 4:** ANOVA results for thermal diffusivity.

Source	df	SS	MS	F value	p-value prob > F	Remarks
Model	3	0.0026	0.0008	5.221	0.0274	Significant
Residual	8	0.0013	0.0001			
Total	11	0.0039				

R-squared: 0.6619, adjusted R-squared: 0.5351.

Table 5 describes the results of ANOVA test for heat flow and shows that the designed model of heat flow was significant (F-value 11.337) and prob > F (p-value 0.0029) respectively. R-squared coefficient explains that 19.05% of the total variables could not be explained by the designed model for heat flow of all developed samples.

**Table 5:** ANOVA results for heat flow.

Source	df	SS	MS	F value	p-value prob > F	Remarks
Model	3	0.0832	0.0277	11.337	0.0029	Significant
Residual	8	0.0195	0.0024			
Total	11	0.1028				

R-squared: 0.8095, adjusted R-squared: 0.7381.

In Table 6, the results demonstrated that the designed ANOVA model for wetting time is statistically significant (F-value 5.644) and prob > F (p-value 0.0224) respectively. The results regarding R-squared coefficient explained that 32.09% of the total variables cannot be explained for wetting time of all samples.

**Table 6:** ANOVA results for wetting time.

Source	df	SS	MS	F value	p-value prob > F	Remarks
Model	3	1478.673	492.891	5.644	0.0224	Significant
Residual	8	698.537	87.317			
Total	11	2177.211				

R-squared: 0.6791, adjusted R-squared: 0.5588.

It was observed that ANOVA test for accumulative one-way transport index is significant (F-value 24.670) and prob > F (p-value 0.00021) as shown in Table 7. R-squared coefficient explained that only 9.76% of the total variables cannot be explained by the designed model for accumulative one-way transport index of all samples.

**Table 7:** ANOVA results for accumulative one-way transport index.

Source	df	SS	MS	F value	p-value prob > F	Remarks
Model	3	501,793.317	167,264.439	24.670	0.00021	Significant
Residual	8	54,240.526	6780.065			
Total	11	556,033.844				

R-squared: 0.9024, adjusted R-squared: 0.8658.

## Conclusion

The objective of this work was to examine the impacts of sonication and nano TiO_2_ coating on thermophysiological properties of fabrics with variation in thickness. For a comprehensive study based on heat and mass transfer, following conclusions were drawn.

- Fabric thickness is a notable parameter that affects thermophysiological properties particularly thermal resistance, thermal diffusivity, wetting time and accumulative one-way transport index. Furthermore, statistically significant results (ANOVA) were obtained for thermal resistance against selected variables of all samples with R^2^ value 0.9651. The result illustrated that sonication and nano TiO_2_ coated fabrics provide significant improvements for thermal insulation. Moreover, in a parallel comparison, the results of thermal resistance of polyester fabric were much lower than cotton fabric.
- A notable consistency was detected for thermal diffusivity of nano TiO_2_ coated and uncoated samples for both fabrics. Fabric thickness performed a metaphorical role in thermal diffusivity. The R^2^ coefficient value was lower for thermal diffusivity due to abnormal distribution of data points.
- The results of heat flow were augmented for both type of fabrics as nano TiO_2_ coated amount and fabric thickness increased. Polyester fabric showed much better results of heat flow than cotton that indicates a higher thermal conductivity of polyester fabric. The results of heat flow were significant (R^2^ 0.8095).
- Fabric structure and surface morphology play critical role in the evaluation of thermophysiological comfort properties. The behaviour of moisture transportation inside fabric structure significantly depends on porosity. The results of wetting time were declined for both type of fabrics as the deposited amount and fabric thickness increased. The decrease of wetting time was a reflection of significantly higher liquid moisture transportation.
- The inspirational findings of this thematic and novel work open a gateway for us to go deeper and extend the procedure for other types of textiles and nanomaterials i.e. polypropylene, polyamide, zinc oxide, copper oxide etc.

## Supplementary information

Supplementary information 1.
