# Supplementary material for: Thermophysiological comfort of sonochemically synthesized nano TiO2 coated woven fabrics
Source: Sci Rep. 2020 Oct 14;10:17204. doi: 10.1038/s41598-020-74357-6 (PMC7560843; doi:10.1038/s41598-020-74357-6)
Supplement: Supplementary file 1 — Supplementary information 1. [file 41598_2020_74357_MOESM1_ESM.docx]

**Thermophysiological comfort of sonochemically synthesized nano TiO_2_ coated woven fabrics**

Muhammad Tayyab Noman^a*^, Michal Petru^a^, Nesrine Amor^b^, Tao Yang^a^, Tariq Mansoor^c^

^a^ Department of Machinery Construction, Institute for Nanomaterials, Advanced Technologies and Innovation (CXI), Studentská 1402/2, 461 17 Liberec 1, Technical University of Liberec, Czech Republic.

^b^ Acoustic Signal Analysis and Processing Group, Faculty of Mechatronics, Informatics and Interdisciplinary Studies, Studentská 1402/2, 461 17 Liberec 1, Technical University of Liberec, Czech Republic.

^c^ Department of Textile Evaluation, Faculty of Textile Engineering, Studentská 1402/2, 461 17 Liberec 1, Technical University of Liberec, Czech Republic.

| 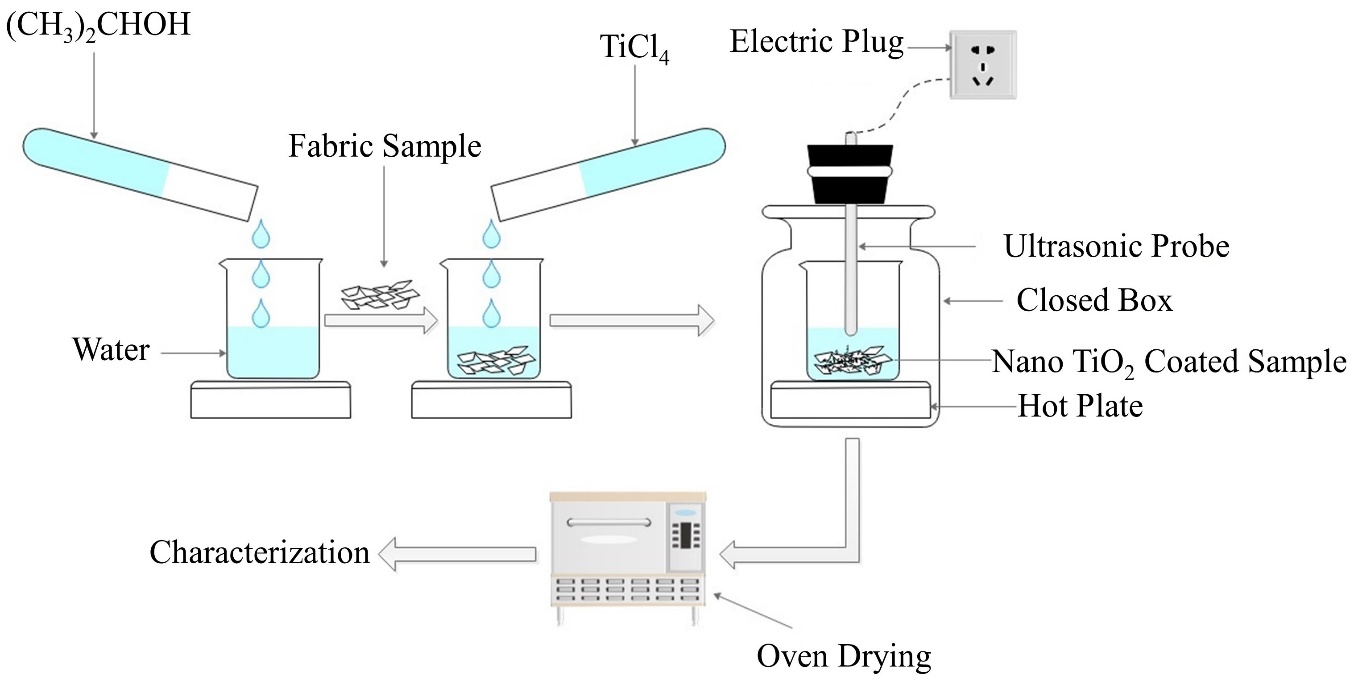  Figure 1S Graphical representation of proposed system and experimental study. |
| --- |

| 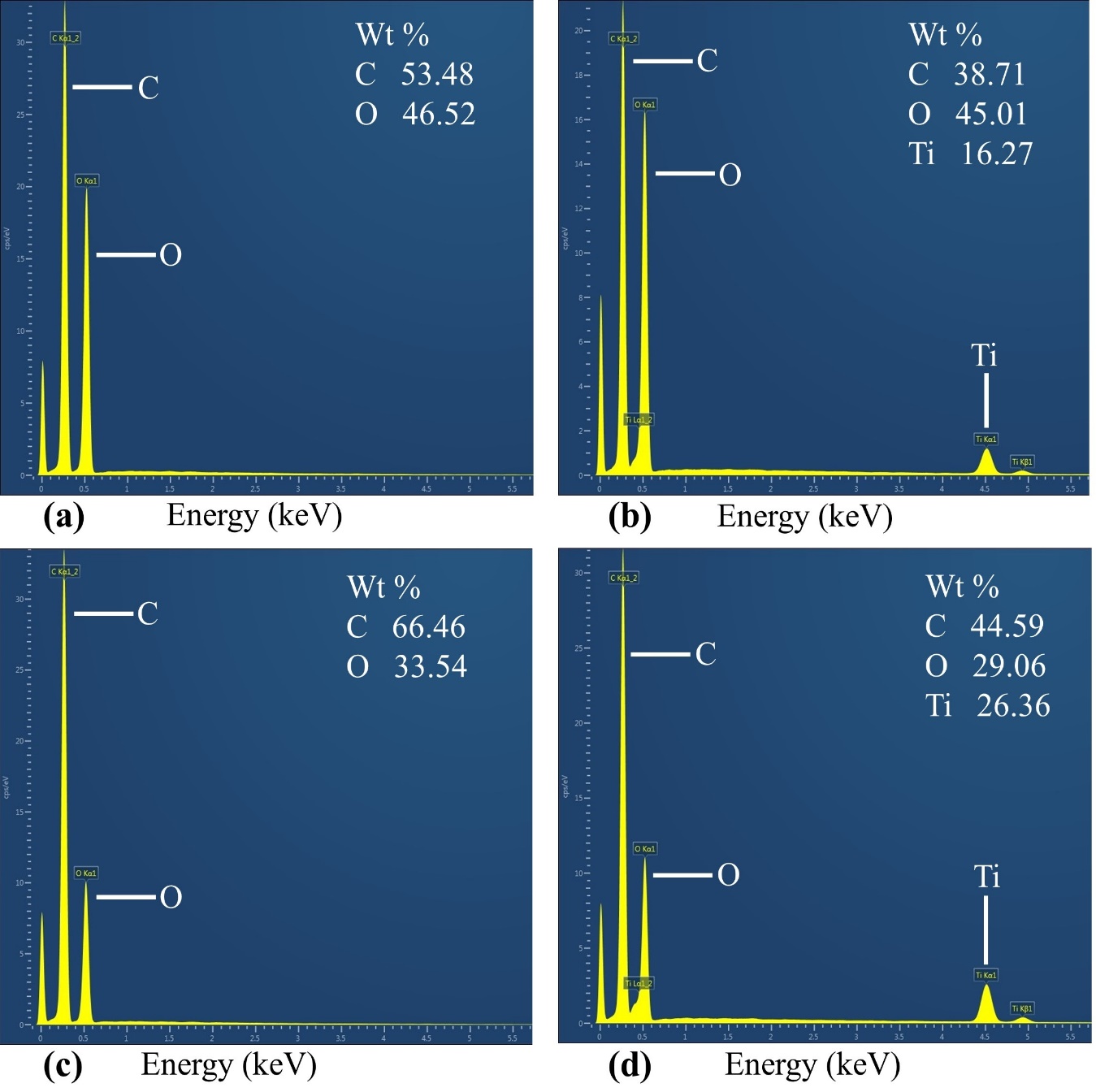  Figure 2S EDX spectra of cotton fabric (a) sample S_1_, and (b) sample S_3_ and for polyester fabric (c) sample S_10_, and (d) sample S_12_ respectively. |
| --- |

EDX spectrum of samples S_3_ and S_12_ confirmed the presence of nano TiO_2_ on cotton and polyester samples respectively whereas no Ti elemental peak was identified in case of untreated sample S_1_ and S_10_ (Figure 2S). Furthermore, the higher weight percentage of Ti element in these samples indicates the higher deposition of nano TiO_2_ over diverse textile substrates that practically explains the benefits of sonication in textile and materials science.

| 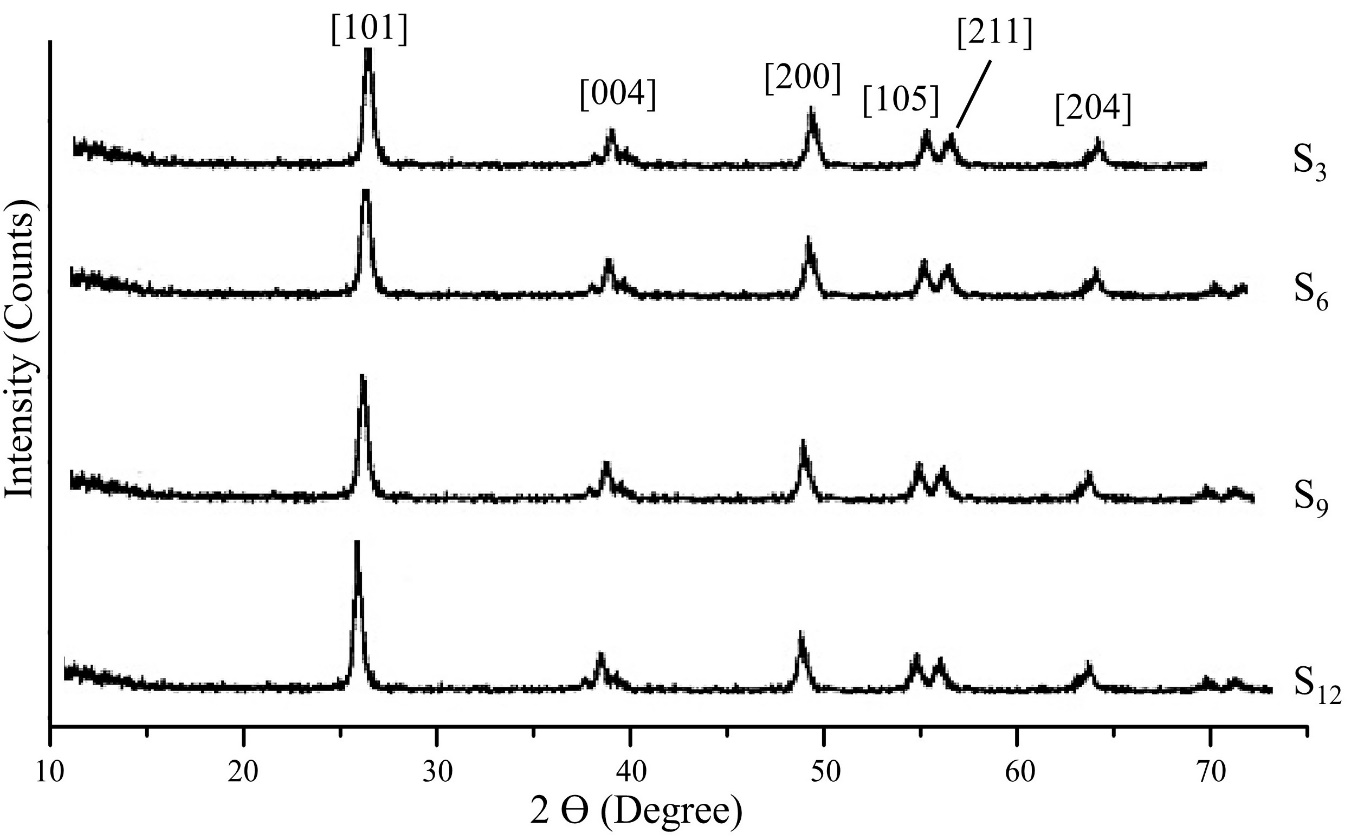  Figure 3S XRD patterns of samples S_3_, S_6_, S_9_ and S_12_ fabricated by sonication. |
| --- |

The collected XRD patterns for all selected samples (S_3_, S_6_, S_9_ and S_12_) confirmed the existence of pure anatase crystals of nano TiO_2_ on both fabrics. The results confirm that all obtained peaks under XRD analysis matched with the International Centre for Diffraction Data (ICDD) Powder Diffraction File (PDF: 00-21-1272). The highest peak for all samples obtained at 2θ = 25.4° is the characteristic crystalline peak for pure anatase TiO_2_ that follows [101] plane reflection as presented in Figure 3S. In addition, a series of crystalline peaks at 2θ = 38°, 48°, 53.8°, 55° and 62° follow [004], [200], [105], [211] and [204] planes respectively. Furthermore, no other phases (impurities) i.e., rutile and brookite, were found during the XRD analysis.
